# Supplementary material for: Evaluation of silver nanoparticles for the prevention of SARS-CoV-2 infection in health workers: In vitro and in vivo
Source: PLoS One. 2021 Aug 19;16(8):e0256401. doi: 10.1371/journal.pone.0256401 (PMC8375774; doi:10.1371/journal.pone.0256401)
Supplement: S1 File — (PDF) [file pone.0256401.s001.pdf]

SS
SECRETARÍA DE SALUD
GOBIERNO DE BAJA CALIFORNIA

ENTIDAD: Instituto de Servicios de Salud Pública del Estado de Baja California.
SECCIÓN: Hospital General Tijuana
SUBSECCIÓN: Departamento de Enseñanza
NUMERO DE OFICIO: 230

Tijuana B.C a 22 de abril de 2020

Dr. Horacio Almanza Reyes.
Investigador principal
Presente.

Estimado Dr. **Almanza Reyes**

Por este conducto informamos a Usted, que el Comité de Ética en Investigación del Hospital General de Tijuana, con dictamen favorable Número CONBIOETICA-02-CEI-001-20170, en la reunión celebrada el día 7 de abril de 2020, habiendo analizado detalladamente ha aprobado la siguiente documentación.

Nombre del Estudio de Investigación: **Evaluación del uso de nanopartículas de plata como producto de higiene bucofaringeo (enjuague bucal) y nasal, por parte del personal de salud que labora en el Hospital General Tijuana expuesto a pacientes con diagnóstico de neumonías atípicas por el SARS-Coronavirus-2.**

Número de Protocolo:
Empresa Patrocinadora del Estudio de Investigación:

ID	Nombre del documento	Versión	Idioma	Fecha
1	Protocolo en extenso	final	Español	7/04/2020
2	Curriculum del investigador principal	N/A	Español	2020
3			Español	

Estamos de acuerdo en que sea Usted, **Dr. Horacio Almanza Reyes.,** el Investigador Principal de dicho protocolo, el cual podrá ser desarrollado bajo las normas internacionales de ética y buena práctica clínica, debiendo reportar los avances hasta el cierre del protocolo

Sin más por el momento, agradezco su amable atención, y envío un cordial saludo.

Dr. Eugenio Oceguera López
Presidente del Comité de Ética en Investigación
Hospital General Tijuana

C c.p. Minutario de Comité Ética en Investigación
