# Supplementary material for: Evaluation of silver nanoparticles for the prevention of SARS-CoV-2 infection in health workers: In vitro and in vivo
Source: PLoS One. 2021 Aug 19;16(8):e0256401. doi: 10.1371/journal.pone.0256401 (PMC8375774; doi:10.1371/journal.pone.0256401)
Supplement: S2 File — (PDF) [file pone.0256401.s002.pdf]

#	COVID 19 + (Positive / Negative)	Gender (Female / Male)	Age (X)	Occupation (Doctor / Nurse / Administrative)	Marital Status (Single / Married / Common Union / Widowed / Divorced)	Smoking habits (Current / Previous / Never)	Seasonal Influenza Vaccine (Yes / No)	Body Mass Index (BMI), (X)	Weight	Height	Type 2 diabetes mellitus (DM), (Yes / No)	Arterial hypertension (HTN), (Yes / No)	Asthma, (Yes / No)	Other diseases	Hand washing before study protocol (X)	Daily gargles before the study protocol (X)	Frequency of upper respiratory tract infections before (last year) of being incorporated into the study protocol (X)	Hand washing during the study protocol (x)	Daily gargles with the oral and nasal hygiene product during the study week (Daily average)	Direct daily application of the oral and nasal hygiene product during the study:	Dayly nasal rinses with the oral and nasal hygiene product during the study week (Daily average)	Time in hours working in the HGT	How many patients were in contact with the diagnosis of atypical pneumonia or COVID-19 disease during the last week (Weekly Average)	What protective equipment was used to prevent infection	Had any symptoms of respiratory tract infections	Adverse reactions reported from using the oral and nasal hygiene product
1	Negative	F	30	Nurse	Single	Never	Yes	26.03	70	1.64	No	No	No	No	20	0	2	20	0.00	2.00	0.00	8	30	PPE	Yes	No
2	Negative	F	32	Nurse	Married	Never	Yes	24.65	60	1.56	No	No	No	No	20	0	1	20	0.11	1.89	1.00	8	90	PPE	No	No
3	Negative	F	49	Nurse	Single	Never	Yes	33.80	100	1.72	No	No	No	No	3	0	2	3	2.44	0.89	1.00	7	0	PPE	No	No
4	Negative	F	28	Nurse	Married	Never	Yes	29.41	85	1.70	No	No	No	No	2	5	1	3	0.11	1.89	1.00	8	341	PPE	Yes	No
5	Negative	F	33	Nurse	Single	Never	No	26.06	78	1.73	Yes	No	No	No	3	1	0	3	3.67	3.44	1.00	12	0	PPE	No	No
6	Negative	M	27	Nurse	Single	Never	Yes	21.28	76	1.89	No	No	No	No	13	0	0	24	2.00	2.00	1.00	8	28	PPE	No	No
7	Negative	F	28	Nurse	Divorced	Never	Yes	26.45	72	1.65	No	Yes	Yes	hypothyroidism	2	0	3	0	0.00	2.44	0.00	8	14	PPE	Yes	No
8	Negative	F	25	Nurse	Single	Never	Yes	24.65	60	1.56	No	No	No	No	3	0	0	3	1.44	0.78	1.00	8	39	PPE	No	No
9	Negative	M	26	Nurse	Married	Never	Yes	28.09	87	1.76	No	No	Yes	No	3	2	0	7	1.11	1.33	0.60	6	128	PPE	No	No
10	Negative	F	26	Nurse	Married	Never	Yes	37.80	92	1.56	No	No	Yes	No	3	0	3	5	2.67	1.44	0.40	8	168	PPE	No	No
11	Negative	F	31	Nurse	Single	Never	No	25.53	67	1.62	No	No	No	No	20	0	1	30	2.00	2.00	0.40	12	65	PPE	No	No
12	Negative	M	29	Nurse	Married	Never	Yes	24.22	70	1.70	No	No	No	No	3	0	3	5	0.00	5.56	0.00	12	46	PPE	Yes	No
13	Negative	F	26	Nurse	Married	Never	Yes	21.83	58	1.63	No	No	No	No	3	2	1	15	2.00	0.00	0.00	8	45	PPE	No	No
14	Negative	M	26	Nurse	Single	Never	No	24.69	80	1.80	No	No	No	No	3	2	0	6	2.00	0.00	0.00	12	45	PPE	No	No
15	Negative	F	44	Administrative	Married	Never	Yes	24.65	60	1.56	No	No	No	No	5	0	1	10	2.00	1.00	0.40	7	45	PPE	No	No
16	Negative	F	27	Nurse	Single	Never	No	21.77	60	1.66	No	No	No	No	10	2	0	3	3.00	3.00	0.60	12	55	PPE	No	No
17	Negative	M	26	Nurse	Single	Never	Yes	30.41	91	1.73	No	No	No	No	0	0	0	20	3.00	0.00	0.00	12	54	PPE	No	No
18	Negative	M	33	Nurse	Single	Never	Yes	25.54	80	1.77	No	No	No	No	10	0	0	15	0.00	2.00	0.00	7	54	PPE	No	No
19	Negative	M	30	Nurse	Married	Never	Yes	27.17	92	1.84	No	No	No	No	15	0	0	40	0.00	1.56	0.00	8	97	PPE	No	No
20	Negative	F	26	Nurse	Married	Never	Yes	20.96	53	1.59	No	No	No	No	3	1	0	3	0.22	1.78	1.00	12	63	PPE	No	No
21	Negative	M	31	Nurse	Common Union	Never	Yes	28.09	88	1.77	No	No	No	hypothyroidism	3	0	0	15	3.00	1.56	0.40	12	182	PPE	No	No
22	Negative	F	33	Nurse	Common Union	Never	Yes	28.91	74	1.60	No	No	No	No	6	2	1	40	0.00	3.00	0.00	12	86	PPE	Yes	No
23	Negative	F	46	Nurse	Divorced	Never	No	22.58	60	1.63	No	No	No	No	8	2	0	40	1.00	0.00	0.00	8	69	PPE	No	No
24	Negative	F	28	Nurse	Single	Never	Yes	21.88	56	1.60	No	No	No	No	3	0	0	3	1.11	1.89	0.60	8	150	PPE	Yes	No
25	Negative	F	44	Nurse	Single	Previous	Yes	34.96	84	1.55	No	No	No	hypothyroidism	3	0	0	10	0.00	3.00	0.00	8	135	PPE	No	No
26	Negative	F	36	Nurse	Married	Never	No	22.31	60	1.64	No	No	No	No	10	0	3	20	3.00	3.00	0.40	7	90	PPE	No	No
27	Negative	F	25	Nurse	Single	Never	Yes	27.56	62	1.50	No	No	No	No	3	1	0	1	1.67	7.33	0.60	8	128	PPE	Yes	No
28	Negative	M	39	Nurse	Single	Never	No	30.42	90	1.72	No	No	No	thrombocytopenia	3	2	1	3	0.22	2.33	0.50	8	256	PPE	No	No
29	Negative	F	23	Nurse	Single	Never	No	22.91	49.5	1.47	No	No	No	No	5	3	1	6	2.78	0.00	0.00	12	190	PPE	No	No
30	Negative	F	26	Nurse	Single	Never	No	27.41	65	1.54	No	No	No	No	10	0	0	10	2.00	0.00	0.00	8	155	PPE	Yes	No
31	Negative	M	63	Nurse	Married	Never	No	24.51	70	1.69	Yes	Yes	No	No	7	0	0	15	0.33	2.00	1.00	7	135	PPE	Yes	No
32	Negative	M	24	Nurse	Married	Previous	No	23.83	61	1.60	No	No	No	No	1	3	0	1	0.00	2.00	0.00	8	175	PPE	No	No
33	Negative	M	25	Nurse	Single	Current	Yes	32.77	105	1.79	No	No	No	No	3	2	2	2	1.00	3.00	0.40	12	180	PPE	No	No
34	Negative	F	26	Nurse	Married	Never	Yes	21.93	52	1.54	No	No	No	No	1	0	0	2	0.00	1.00	0.00	6	180	PPE	No	No
35	Negative	M	30	Nurse	Married	Never	Yes	24.22	70	1.70	No	No	No	No	10	0	0	25	0.00	2.00	0.00	8	180	PPE	No	No
36	Negative	F	22	Nurse	Single	Never	Yes	29.38	80	1.65	No	No	No	No	3	0	0	20	0.00	2.11	0.00	8	167	PPE	No	No
37	Negative	M	58	Doctor	Married	Never	No	22.86	70	1.75	No	No	No	No	20	0	2	20	2.00	2.00	0.50	7	180	PPE	No	No
38	Negative	F	26	Nurse	Single	Previous	Yes	34.53	94	1.65	No	No	No	No	3	0	2	5	2.00	0.00	0.00	36	398	PPE	No	No
39	Negative	M	40	Doctor	Married	Never	Yes	28.41	90	1.78	No	No	No	No	15	0	0	30	0.00	1.00	0.00	8	225	PPE	No	No
40	Negative	F	26	Nurse	Single	Current	Yes	21.04	56.6	1.64	No	No	No	No	3	2	2	12	0.00	2.00	0.00	12	198	PPE	No	No
41	Negative	F	30	Nurse	Single	Never	Yes	24.97	60	1.55	No	No	No	No	20	3	2	30	2.33	0.00	0.00	12	151	PPE	Yes	No
42	Negative	F	27	Nurse	Single	Previous	No	24.22	70	1.70	No	No	No	No	6	3	1	15	2.00	0.00	0.00	12	216	PPE	No	No
43	Negative	F	30	Nurse	Married	Never	Yes	24.84	62	1.58	No	No	No	No	5	0	0	12	2.00	0.00	0.00	12	199	PPE	No	No
44	Negative	F	29	Nurse	Single	Never	Yes	29.41	85	1.70	No	No	No	No	4	1	0	12	3.00	3.00	1.00	12	184	PPE	No	No
45	Negative	F	27	Nurse	Single	Never	Yes	22.21	59	1.63	No	No	No	No	15	0	0	40	0.00	2.22	0.00	8	228	PPE	Yes	No
46	Negative	F	36	Nurse	Married	Never	Yes	23.80	64	1.64	No	No	No	No	1	0	2	0	0.11	1.89	1.00	12	231	PPE	No	No
47	Negative	F	47	Nurse	Married	Never	Yes	22.31	66	1.72	No	No	No	No	3	0	1	10	2.33	2.67	0.40	7	270	PPE	No	No
48	Negative	F	33	Nurse	Married	Never	Yes	21.36	50	1.53	No	No	No	No	6	0	0	12	3.00	4.00	0.40	12	270	PPE	No	No
49	Negative	M	31	Nurse	Married	Never	Yes	30.12	82	1.65	No	No	No	No	30	0	2	30	1.00	0.00	0.00	12	270	PPE	No	No
50	Negative	M	26	Nurse	Single	Never	Yes	27.34	70	1.60	No	No	No	No	3	2	0	3	1.00	1.00	1.00	8	345	PPE	No	No
51	Negative	F	26	Nurse	Single	Never	Yes	26.31	60	1.51	No	No	No	No	3	2	2	3	2.22	0.00	0.00	12	550	PPE	No	No
52	Negative	M	26	Nurse	Single	Never	Yes	20.83	66	1.78	No	No	No	No	3	2	0	3	0.22	2.00	1.00	8	388	PPE	Yes	No
53	Negative	M	22	Nurse	Single	Never	Yes	25.69	76	1.72	No	No	No	No	3	2	0	4	0.00	3.78	0.00	8	545	PPE	Yes	No
54	Negative	F	26	Nurse	Single	Never	No	26.22	74	1.68	No	No	No	No	8	0	3	20	2.00	0.00	0.00	12	226	PPE	Yes	No
55	Negative	F	28	Nurse	Single	Never	Yes	31.22	85	1.65	No	No	No	No	3	2	1	2	1.00	2.67	1.00	8	495	PPE	Yes	No
56	Negative	M	25	Nurse	Married	Never	Yes	35.91	105	1.71	No	No	No	No	7	2	0	20	0.00	3.00	0.00	12	455	PPE	No	No
57	Negative	F	24	Nurse	Single	Never	Yes	31.24	77	1.57	No	No	No	No	3	3	0	6	3.00	3.00	0.40	8	860	PPE	No	No
58	Negative	M	47	Doctor	Married	Never	Yes	29.99	106	1.88	No	No	No	No	10	0	1	20	0.00	4.00	0.00	7	390	PPE	No	No
59	Negative	M	73	Doctor	Married	Never	Yes	29.73	90	1.74	No	Yes	No	No	3	0	0	6	2.00	4.00	1.00	8	270	PPE	No	No
60	Negative	F	44	Nurse	Married	Never	Yes	29.73	79	1.63	No	No	No	No	5	5	0	3	3.00	3.00	1.00	8	135	PPE	No	No
61	Negative	F	38	Nurse	Married	Never	Yes	21.88	56	1.60	No	No	No	No	0	0	0	2	0.22	1.78	1.00	12	371	PPE	No	No
62	Negative	F	24	Nurse	Single	Never	Yes	27.24	68	1.58	No	No	No	No	3	0	2	20	0.00	14.00	0.00	12	330	PPE	Yes	No
63	Negative	F	62	Nurse	Divorced	Never	No	22.72	56	1.57	No	No	No	No	10	0	3	15	2.00	1.00	0.40	7	36	PPE	No	No
64	Negative	F	32	Administrative	Married	Never	No	23.62	62	1.62	No	No	No	No	8	0	0	20	0.00	4.00	0.00	7	36	PPE	No	No
65	Negative	M	35	Nurse	Married	Never	No	27.34	79	1.70	No	No	No	No	3	1	3	2	3.00	0.00	0.00	7	36	PPE	No	No
66	Negative	F	41	Nurse	Divorced	Never	No	26.03	70	1.64	No	No	No	No	3	0	2	3	2.00	0.00	0.00	7	36	PPE	No	No
67	Negative	M	64	Administrative	Divorced	Never	No	28.84	72	1.58	No	No	No	No	3	0	2	8	2.00	0.00	0.00	7	36	PPE	No	No
68	Negative	F	65	Administrative	Married	Previous	No	29.38	80	1.65	No	Yes	No	No	4	0	2	10	2.00	0.00	0.00	7	36	PPE	No	No
69	Negative	F	52	Administrative	Married	Never	No	25.78	58	1.50	Yes	Yes	No	No	3	2	3	3	2.00	0.00	0.00	7	36	PPE	No	No
70	Negative	F	61	Nurse	Married	Never	No	23.44	60	1.60	No	No	No	No	3	1	1	6	2.00	0.00	0.00	7	36	PPE	No	No
71	Negative	F	30	Nurse	Common Union	Never	No	24.44	55	1.50	No	No	No	No	3	0	0	10	2.00	0.00	0.00	7	36	PPE	No	No
72	Negative	M	30	Administrative	Common Union	Never	No	24.22	70	1.70	No	No	No	No	3	1	0	2	2.00	0.00	0.00	7	36	PPE	No	No

#	COVID 19 + (Positive / Negative)	Gender (Female / Male)	Age (X)	Occupation (Doctor / Nurse / Administrative)	Marital Status (Single / Married / Common Union / Widowed / Divorced)	Smoking habits (Current / Previous / Never)	Seasonal Influenza Vaccine (Yes / No)	Body Mass Index (BMI), (X)	Weight	Height	Type 2 diabetes mellitus (DM), (Yes / No)	Arterial hypertension (HTN), (Yes / No)	Asthma, (Yes / No)	Other diseases	Hand washing before study protocol (X)	Daily gargles before the study protocol (X)	Frequency of upper respiratory tract infections before (last year) of being incorporated into the study protocol (X)	Hand washing during the study protocol (x)	Daily gargles with the oral and nasal hygiene product during the study week (Daily average)	Direct daily application of the oral and nasal hygiene product during the study:	Dayly nasal rinses with the oral and nasal hygiene product during the study week (Daily average)	Time in hours working in the HGT	How many patients were in contact with the diagnosis of atypical pneumonia or COVID-19 disease during the last week (Weekly Average)	What protective equipment was used to prevent infection	Had any symptoms of respiratory tract infections	Adverse reactions reported from using the oral and nasal hygiene product
73	Negative	M	25	Nurse	Single	Never	Yes	23.46	67	1.69	No	No	No	No	3	2	0	4	1.78	0.44	0.60	7	0	PPE	No	No
74	Negative	F	28	Nurse	Married	Never	Yes	24.00	71	1.72	No	No	No	No	2	2	0	4	8.00	8.00	1.00	8	66	PPE	No	No
75	Negative	F	36	Nurse	Single	Never	Yes	24.22	62	1.60	No	No	Yes	No	3	2	2	3	1.11	0.22	1.00	8	216	PPE	Yes	No
76	Negative	F	25	Nurse	Single	Previous	Yes	24.09	68	1.68	No	No	No	No	20	2	3	20	12.78	0.00	0.00	12	334	PPE	No	No
77	Negative	F	24	Nurse	Single	Never	Yes	20.82	56	1.64	No	No	No	No	3	1	1	1	1.00	2.78	0.40	8	54	PPE	No	No
78	Negative	M	32	Doctor	Married	Never	Yes	24.49	75	1.75	No	No	No	No	3	1	2	8	3.00	3.00	1.00	7	270	PPE	No	No
79	Negative	F	30	Nurse	Married	Never	No	24.89	56	1.50	No	No	No	No	8	2	2	10	4.00	28.00	0.40	7	81	PPE	No	No
80	Negative	F	20	Nurse	Single	Never	Yes	20.00	45	1.50	No	No	No	No	5	2	0	7	5.00	35.00	1.00	7	135	PPE	No	No
81	Negative	M	60	Doctor	Single	Never	No	25.24	63	1.58	No	No	No	No	6	2	1	15	5.00	33.67	1.00	8	235	PPE	No	No
82	Negative	F	65	Administrative	Widowed	Never	No	26.67	60	1.50	No	No	No	No	7	2	1	10	6.78	29.89	0.60	15	135	PPE	No	No
83	Negative	M	52	Doctor	Single	Previous	No	22.84	66	1.70	No	No	No	No	6	2	0	10	3.00	3.00	0.60	8	63	PPE	No	No
84	Negative	F	20	Nurse	Single	Never	No	18.29	48	1.62	No	No	No	No	4	0	0	10	3.00	4.00	0.60	8	65	PPE	No	No
85	Negative	F	58	Nurse	Married	Previous	No	29.33	66	1.50	No	No	No	No	7	0	0	10	3.00	3.00	1.00	8	86	PPE	No	No
86	Negative	F	62	Nurse	Single	Never	Yes	28.54	60	1.45	No	No	No	No	3	0	0	10	3.00	3.00	1.00	8	105	PPE	No	No
87	Negative	M	49	Administrative	Married	Never	Yes	24.61	63	1.60	No	No	No	No	3	0	0	6	3.00	3.00	0.60	8	169	PPE	No	No
88	Negative	M	61	Doctor	Widowed	Never	No	22.49	65	1.70	Yes	Yes	No	No	3	0	0	10	3.00	3.00	0.60	8	106	PPE	No	No
89	Negative	M	43	Administrative	Married	Never	No	23.78	72	1.74	No	No	No	No	3	0	0	8	3.00	3.00	0.40	8	86	PPE	No	No
90	Negative	F	63	Administrative	Married	Never	No	22.22	50	1.50	No	No	No	No	3	0	0	10	3.00	3.00	0.40	8	89	PPE	No	No
91	Negative	M	47	Doctor	Married	Never	No	26.12	80	1.75	No	No	No	No	3	0	0	10	3.56	3.56	0.40	8	231	PPE	No	No
92	Positive.	M	30	Nurse	Single	Never	Yes	27.08	82	1.74	No	No	No	No	3	2	0	3	9.56	4.78	1.00	12	2	PPE	No	No
93	Positive.	F	27	Nurse	Single	Never	Yes	28.26	76	1.64	No	No	No	No	20	0	3	20	1.50	0.67	1.00	12	20	PPE	Yes	No
94	Negative	F	33	Nurse	Married	Never	Yes	23.44	60	1.60	No	No	No	No	5	4	0	5	4.00	6.89	0.40	7	270	PPE	Yes	No
95	Negative	M	32	Nurse	Married	Never	Yes	24.39	73	1.73	No	No	No	No	2	0	0	2	2.78	0.00	0.00	12	448	PPE	No	No
96	Negative	F	30	Nurse	Married	Never	Yes	28.23	75	1.63	No	No	No	No	0	0	1	0	0.44	1.56	1.00	12	318	PPE	No	No
97	Negative	F	37	Nurse	Married	Never	Yes	28.44	72.8	1.60	No	No	No	No	3	1	0	2	0.00	2.00	0.00	6	63	PPE	No	No
98	Negative	M	32	Nurse	Single	Never	Yes	27.68	80	1.70	No	No	No	No	3	0	0	0	0.44	2.00	0.40	12	404	PPE	No	No
99	Negative	M	32	Nurse	Married	Never	Yes	27.04	80	1.72	No	No	No	No	3	0	0	0	0.00	2.00	0.00	12	456	PPE	No	No
100	Negative	F	30	Nurse	Single	Never	Yes	30.11	80	1.63	No	No	No	No	3	0	0	0	1.11	0.89	1.00	12	248	PPE	No	No
101	Negative	M	30	Nurse	Single	Never	Yes	28.40	85	1.73	No	No	No	No	0	0	0	0	0.00	3.00	0.00	12	453	PPE	No	No
102	Negative	M	23	Nurse	Single	Never	Yes	23.88	69	1.70	No	No	No	No	6	2	2	8	10.00	2.89	0.40	12	350	PPE	No	No
103	Negative	F	29	Nurse	Single	Never	Yes	36.59	107	1.71	No	No	No	No	5	2	0	1	1.00	1.00	0.40	12	72	PPE	Yes	No
104	Negative	F	44	Nurse	Single	Never	Yes	32.67	79.5	1.56	No	No	No	No	80	4	0	80	3.11	0.00	0.00	12	392	PPE	No	No
105	Negative	F	62	Administrative	Common Union	Never	Yes	23.71	63	1.63	No	Yes	No	No	10	0	0	30	2.00	0.00	0.00	8	0	PPE	No	No
106	Negative	F	30	Nurse	Common Union	Never	Yes	24.56	59	1.55	No	No	No	No	2	0	2	10	2.00	2.00	1.00	12	90	PPE	No	No
107	Negative	F	32	Nurse	Married	Never	Yes	34.63	80	1.52	No	No	No	No	5	0	4	20	2.00	0.00	0.00	8	44	PPE	No	No
108	Negative	F	32	Administrative	Single	Never	Yes	32.79	85	1.61	No	No	No	No	6	2	2	25	2.00	0.00	0.00	8	9	PPE	Yes	No
109	Negative	F	33	Administrative	Married	Never	No	24.61	63	1.60	No	No	No	No	5	1	3	10	1.00	1.00	0.60	7	20	PPE	No	No
110	Negative	F	32	Administrative	Common Union	Never	No	24.34	60	1.57	No	No	No	No	5	2	2	30	2.00	2.00	0.40	7	0	PPE	No	No
111	Negative	M	41	Nurse	Common Union	Never	No	34.19	117	1.85	No	No	Yes	No	5	1	1	20	3.00	3.00	0.40	8	90	PPE	No	No
112	Negative	F	40	Nurse	Single	Never	No	0.01	135	158	No	No	No	No	5	2	3	15	1.00	0.00	0.00	7	0	PPE	No	No
113	Negative	M	36	Nurse	Single	Never	Yes	21.47	65	1.74	No	No	No	No	3	1	0	-	3.00	1.00	0.40	12	216	PPE	No	No
114	Negative	F	26	Nurse	Single	Never	No	18.36	47	1.6	No	No	No	No	3	1	1	6	1.00	1.00	0.40	12	216	PPE	No	No

0.6
0.4
