# Supplementary material for: Evaluation of silver nanoparticles for the prevention of SARS-CoV-2 infection in health workers: In vitro and in vivo
Source: PLoS One. 2021 Aug 19;16(8):e0256401. doi: 10.1371/journal.pone.0256401 (PMC8375774; doi:10.1371/journal.pone.0256401)
Supplement: S3 File — (PDF) [file pone.0256401.s003.pdf]

#	COVID 19 + (Positive / Negative)	Gender (Female / Male)	Age (X)	Occupation (Doctor / Nurse / Administrative)	Marital Status (Single / Married / Common Union / Viudo / Divorced)	Smoking habits (Current / Previous / Never)	Seasonal Influenza Vaccine (Yes / No)	Body Mass Index (BMI), (X)	Weight	Height	Type 2 diabetes mellitus (DM), (Yes / No)	Arterial hypertenYeson (HTN), (Yes / No)	Asthma, (Yes / No)	Other diseases	Hand washing before study protocol (X)	Daily gargles before the study protocol (X)	Frequency of upper respiratory tract infections before (last year) of being incorporated into the study protocol (X)	Hand washing during the study protocol (x)	Daily gargles with a convencional mouthwash during the study week (Daily average)	Direct daily application of the oral and nasal hygiene product during the study:	Dayly nasal rinses with the oral and nasal hygiene product during the study week (Daily average)	Time in hours working in the HGT	How many patients were in contact with the diagnosis of atypical pneumonia or COVID-19 disease during the last week (Weekly Average)	What protective equipment was used to prevent infection	Had any symptoms of respiratory tract infections
1	Negative	M	27	Doctor	Single	Current	Yes	23.8	73	1.75	No	No	No	No	3	0	2	3	0	0	0	8	108	PPE	No
2	Negative	M	45	Nurse	Single	Never	Yes	27.5	75	1.65	No	No	No	No	10	0	2	10	0	0	0	12	410	PPE	No
3	Negative	F	33	Nurse	Single	Never	Yes	28.5	73	1.60	No	No	No	No	20	3	2	20	2	0	0	12	216	PPE	No
4	Negative	M	30	Nurse	Single	Never	Yes	39.4	110	1.67	No	No	No	No	0	0	0	0	0	0	0	12	189	PPE	No
5	Negative	F	45	Nurse	Married	Current	No	33.7	82	1.56	No	Yes	No	No	15	2	0	15	2	0	0.2	12	186	PPE	No
6	Negative	F	48	Nurse	Single	Never	Yes	24.1	58	1.55	No	No	No	No	15	3	0	15	0	0	0	12	125	PPE	No
7	Negative	F	30	Nurse	Married	Never	Yes	39.2	116	1.72	No	No	No	No	15	2	3	15	2	0	0	12	165	PPE	No
8	Negative	M	53	Nurse	Married	Current	Yes	30.1	87	1.70	No	No	No	No	10	0	0	15	0	0	0	8	540	PPE	No
9	Negative	F	32	Administrative	Single	Never	Yes	23.4	60	1.60	No	No	No	No	2	0	0	4	2	0	0	12	0	PPE	No
10	Negative	M	50	Administrative	Married	Previous	Yes	33.9	98	1.70	Yes	No	No	No	4	0	0	0	0	0	0	7	81	PPE	No
11	Negative	M	56	Nurse	Married	Previous	Yes	30.4	76	1.58	No	No	No	No	10	0	0	6	0	0	0	6.5	216	PPE	No
12	Negative	F	29	Administrative	Common Union	Never	No	29.0	80	1.66	No	No	No	pothyroidis	10	0	0	30	1	0	0.2	12	86	PPE	No
13	Negative	M	28	Nurse	Single	Never	Yes	23.0	68	1.72	No	No	No	No	10	0	1	20	0	0	0	8	240	PPE	No
14	Negative	F	26	Nurse	Single	Never	Yes	29.1	69	1.54	No	No	No	No	20	0	1	20	0	0	0	7	225	PPE	No
15	Negative	M	29	Nurse	Single	Never	Yes	24.9	72	1.70	No	Yes	No	No	3	0	2	20	0	0	0	8	240	PPE	Yes
16	Negative	M	34	Administrative	Married	Never	Yes	30.9	100	1.80	No	No	No	pothyroidis	0	0	0	0	2	0	0	6.5	270	PPE	No
17	Negative	M	25	Nurse	Single	Never	Yes	23.7	70	1.72	No	No	No	No	2	1	1	2	0	0	0	12	63	PPE	No
18	Negative	F	51	Administrative	Married	Never	Yes	25.6	68	1.63	No	No	No	pothyroidis	8	2	1	12	0	0	0	6.5	180	PPE	No
19	Negative	M	45	Doctor	Married	Never	No	31.2	93.5	1.73	Yes	No	No	No	10	0	1	10	0	0	0	8	270	PPE	Yes
20	Negative	F	42	Administrative	Divorced (a)	Current	Yes	27.6	76	1.66	No	No	No	No	20	0	0	20	0	0	0	7	135	PPE	No
21	Negative	F	49	Nurse	Married	Never	Yes	25.3	64	1.59	No	No	No	No	3	0	0	20	0	0	0	7	180	PPE	No
22	Negative	F	33	Administrative	Single	Never	Yes	27.2	75	1.66	No	No	No	No	20	0	0	10	0	0	0	6.5	450	PPE	No
23	Negative	F	28	Nurse	Single	Current	Yes	29.6	73	1.57	No	No	No	No	1	3	0	1	4	0	0.2	12	54	PPE	Yes
24	Negative	F	23	Nurse	Single	Never	Yes	23.9	65	1.65	No	No	No	No	3	0	0	10	0	0	0	12	63	PPE	No
25	Negative	F	26	Nurse	Single	Never	Yes	27.1	71	1.62	No	No	No	No	20	0	2	20	0	0	0	12	123	PPE	Yes
26	Negative	F	23	Nurse	Single	Never	No	21.6	61	1.68	No	No	No	No	0	0	0	2	0	0	0	12	54	PPE	No
27	Negative	M	23	Nurse	Single	Never	Yes	35.4	84	1.54	No	No	No	No	3	0	0	10	0	0	0	12	135	PPE	No
28	Negative	M	48	Nurse	Married	Never	Yes	30.4	76	1.58	No	No	No	No	10	0	0	30	0	0	0	12	0	PPE	Yes
29	Negative	M	46	Nurse	Single	Never	No	35.1	98	1.67	No	No	No	No	10	0	1	10	4	0	0	12	450	PPE	No
30	Negative	F	37	Nurse	Single	Never	No	26.4	65	1.57	No	No	No	No	10	0	1	20	0	0	0.2	12	30	PPE	No
31	Negative	F	45	Nurse	Common Union	Never	No	32.0	73	1.51	No	Yes	No	pothyroidis	10	0	0	20	0	0	0	12	50	PPE	Yes
32	Negative	M	32	Nurse	Single	Never	Yes	28.7	78	1.65	No	No	No	gastritis	25	2	1	25	0	0	0	12	147	PPE	No
33	Negative	F	43	Nurse	Common Union	Never	Yes	26.7	70	1.62	No	No	No	No	8	0	0	10	2	0	0	12	720	PPE	No
34	Negative	F	46	Nurse	Married	Never	Yes	31.3	80	1.60	No	No	No	onic migra	10	0	0	10	0	0	0	12	468	PPE	No
35	Negative	F	24	Nurse	Single	Never	Yes	25.2	80	1.78	No	No	No	No	10	0	3	10	0	0	0	12	180	PPE	No
36	Negative	F	30	Nurse	Single	Never	Yes	22.4	58	1.61	No	No	No	No	10	0	0	10	2	0	0	12	0	PPE	No
37	Negative	F	48	Administrative	Single	Never	Yes	34.4	88	1.60	No	No	No	Hepatitis	3	0	0	10	0	0	0	6.5	0	PPE	No
38	Negative	F	28	Nurse	Single	Previous	Yes	25.6	64	1.58	No	No	No	No	10	0	3	30	0	0	0	12	180	PPE	No
39	Negative	F	37	Nurse	Married	Never	Yes	28.4	72.8	1.60	No	No	No	No	3	1	2	2	0	0	0	12	0	PPE	Yes
40	Negative	F	28	Nurse	Married	Never	Yes	33.6	87	1.61	No	No	No	obesity	30	0	2	0	4	0	0	8	216	PPE	Yes
41	Negative	F	25	Nurse	Common Union	Current	Yes	18.4	50	1.65	No	No	No	No	10	2	2	30	0	0	0	12	72	PPE	No
42	Negative	F	26	Nurse	Single	Never	Yes	33.1	90	1.65	No	No	No	No	10	0	0	5	0	0	0	12	154	PPE	No
43	Negative	F	24	Nurse	Single	Never	Yes	28.6	71.4	1.58	No	No	No	No	10	0	3	15	0	0	0	12	163	PPE	No
44	Negative	M	23	Nurse	Single	Never	Yes	29.4	85	1.70	No	No	No	No	10	0	1	4	0	0	0	12	107	PPE	Yes
45	Negative	M	28	Nurse	Single	Never	Yes	51.5	154	1.73	No	No	No	No	3	0	0	3	0	0	0	8	198	PPE	No
46	Negative	F	29	Nurse	Married	Never	Yes	25.1	66	1.62	No	No	Yes	No	0	0	0	0	0	0	0	8	252	PPE	No
47	Negative	F	23	Nurse	Single	Never	Yes	18.6	43	1.52	No	No	No	No	3	0	0	3	0	0	0	8	198	PPE	No
48	Negative	M	28	Nurse	Single	Current	Yes	24.8	70	1.68	No	No	No	No	0	0	1	20	2	0	0	8	170	PPE	No
49	Negative	F	28	Nurse	Single	Never	Yes	31.1	92	1.72	No	No	No	No	8	3	1	8	0	0	0	12	36	PPE	No
50	Negative	M	24	Nurse	Single	Current	Yes	22.2	56	1.59	No	No	No	No	0	0	2	0	0	0	0	8	224	PPE	No
51	Negative	F	40	Nurse	Married	Never	Yes	34.8	88	1.59	Yes	Yes	No	No	10	0	3	20	0	0	0	8	252	PPE	Yes
52	Negative	M	32	Nurse	Single	Current	Yes	25.2	72	1.69	No	No	No	No	9	2	2	25	0	0	0	12	216	PPE	No
53	Negative	F	34	Nurse	Married	Never	Yes	28.2	74.8	1.63	Yes	No	No	No	10	2	1	20	0	0	0	12	216	PPE	No
54	Negative	F	39	Nurse	Married	Never	Yes	25.0	57	1.51	No	No	No	matoid art	30	1	2	0	0	0	0	8	216	PPE	Yes
55	Negative	F	34	Nurse	Single	Never	Yes	27.7	64	1.52	No	No	No	No	10	0	1	20	0	0	0	12	98	PPE	No
56	Negative	F	29	Nurse	Married	Never	Yes	28.0	80	1.69	No	No	No	No	4	0	0	0	0	0	0.2	8	157	PPE	No
57	Negative	F	37	Nurse	Married	Never	Yes	31.2	78	1.58	No	No	No	No	10	0	0	3	0	0	0	12	27	PPE	No
58	Negative	F	25	Nurse	Single	Never	Yes	33.3	69	1.44	No	No	No	No	5	0	0	2	0	0	0	12	216	PPE	No
59	Negative	F	27	Nurse	Married	Never	Yes	27.9	77	1.66	No	No	No	No	10	0	1	20	0	0	0.2	12	216	PPE	No
60	Negative	F	29	Nurse	Single	Never	Yes	38.3	103	1.64	No	No	Yes	No	10	0	1	0	0	0	0.2	8	160	PPE	No
61	Negative	F	21	Nurse	Single	Never	Yes	30.3	95	1.77	No	No	No	No	0	0	3	30	0	0	0	8	252	PPE	No
62	Negative	F	30	Nurse	-	Never	Yes	-	-	-	No	No	No	No	-	1	1	-	0	0	0	8	0	PPE	Yes
63	Negative	F	30	Nurse	-	Never	Yes	-	-	-	No	No	No	No	-	2	1	-	0	0	0.4	8	0	PPE	Yes
64	Negative	M	27	Nurse	Single	Previous	Yes	34.6	100	1.70	No	No	No	No	5	3	2	30	0	0	0.4	12	63	PPE	No
65	Negative	M	23	Nurse	Single	Never	Yes	24.1	72	1.73	No	No	No	No	10	0	3	20	0	0	0	12	63	PPE	No
66	Negative	F	39	Nurse	Divorced (a)	Current	Yes	25.0	64	1.60	No	No	No	No	10	0	0	20	0	0	0	12	198	PPE	No
67	Negative	M	26	Nurse	Single	Never	Yes	23.6	80	1.84	No	No	No	No	3	3	0	2	0	0	0	12	54	PPE	No
68	Negative	M	29	Nurse	Common Union	Previous	Yes	28.7	85	1.72	No	No	Yes	No	0	0	3	0	0	0	0	12	0	PPE	Yes
69	Negative	F	46	Nurse	Single	Never	Yes	29.1	70	1.55	No	No	No	No	10	3	1	4	0	0	0	12	216	PPE	No
70	Negative	F	31	Nurse	Single	Never	Yes	27.3	70	1.60	No	No	No	No	10	0	0	20	0	0	0.2	12	207	PPE	No
71	Negative	M	33	Nurse	Married	Never	Yes	29.7	92	1.76	No	No	No	No	10	0	2	10	0	0	0	12	54	PPE	No
72	Negative	M	43	Nurse	Single	Never	Yes	27.8	73	1.62	No	No	No	No	10	0	2	20	2	0	0	12	108	PPE	No
73	Negative	F	26	Nurse	Single	Never	No	24.4	61	1.58	No	No	No	No	3	0	0	20	2	0	0	12	180	PPE	No
74	Negative	F	29	Nurse	Married	Never	Yes	30.1	87	1.70	No	No	No	No	10	0	0	20	0	0	0	12	729	PPE	No

#	COVID 19 + (Positive / Negative)	Gender (Female / Male)	Age (X)	Occupation (Doctor / Nurse / Administrative)	Marital Status (Single / Married / Common Union / Viudo / Divorced)	Smoking habits (Current / Previous / Never)	Seasonal Influenza Vaccine (Yes / No)	Body Mass Index (BMI), (X)	Weight	Height	Type 2 diabetes mellitus (DM), (Yes / No)	Arterial hypertenYeson (HTN), (Yes / No)	Asthma, (Yes / No)	Other diseases	Hand washing before study protocol (X)	Daily gargles before the study protocol (X)	Frequency of upper respiratory tract infections before (last year) of being incorporated into the study protocol (X)	Hand washing during the study protocol (x)	Daily gargles with a convencional mouthwash during the study week (Daily average)	Direct daily application of the oral and nasal hygiene product during the study:	Dayly nasal rinses with the oral and nasal hygiene product during the study week (Daily average)	Time in hours working in the HGT	How many patients were in contact with the diagnosis of atypical pneumonia or COVID-19 disease during the last week (Weekly Average)	What protective equipment was used to prevent infection	Had any symptoms of respiratory tract infections
**75**	Negative	M	32	Nurse	Common Union	Never	Yes	30.0	93	1.76	No	No	No	No	10	0	0	20	0	0	0	12	222	PPE	No
**76**	Negative	M	28	Nurse	Single	Never	Yes	32.7	100	1.75	No	No	No	Rhinitis	3	4	1	20	2	0	0	12	600	PPE	No
**77**	Negative	F	36	Nurse	Married	Never	Yes	32.0	82	1.60	No	No	No	No	10	0	2	20	0	0	0.2	12	206	PPE	No
**78**	Negative	F	43	Nurse	Married	Never	Yes	24.2	70	1.70	No	No	No	No	20	0	2	40	0	0	0	12	216	PPE	No
**79**	Negative	F	22	Nurse	Single	Never	Yes	33.0	84.4	1.60	No	No	No	No	20	0	2	20	0	0	0	12	140	PPE	No
**80**	Negative	F	29	Nurse	Married	Never	Yes	27.4	65	1.54	No	No	No	plitis, bronc	20	0	2	20	0	0	0	12	207	PPE	No
**81**	Negative	F	24	Nurse	Single	Never	Yes	34.0	87	1.60	No	No	No	No	10	0	1	20	0	0	0	12	153	PPE	No
**82**	Negative	F	42	Nurse	Married	Never	Yes	33.3	80	1.55	No	No	No	No	3	0	0	3	0	0	0	12	195	PPE	No
**83**	Negative	M	40	Nurse	Married	Never	No	30.1	81	1.64	No	No	No	No	10	3	1	20	2	0	0	12	198	PPE	No
**84**	Negative	F	49	Nurse	Single	Never	No	30.0	72	1.55	No	No	No	No	0	0	0	0	0	0	0	12	216	PPE	No
**85**	Positive	M	32	Nurse	Common Union	Previous	Yes	30.80	89	1.7	No	No	No	No	15	0	2	15	0	0	0.2	8	24	PPE	Yes
**86**	Positive	F	40	Administrative	Married	Never	Yes	27.03	60	1.49	No	No	No	No	18	0	0	15	2	0	0	12	5	PPE	Yes
**87**	Positive	M	22	Nurse	Single	Current	Yes	28.90	88.5	1.75	No	No	No	No	25	0	0	25	0	0	0	8	10	PPE	Yes
**88**	Positive	F	26	Nurse	Single	Never	No	24.22	70	1.7	No	No	No	No	10	0	0	20	0	0	0	8	6	PPE	Yes
**89**	Positive	F	48	Nurse	Single	Never	Yes	27.34	70	1.6	Yes	No	No	No	10	2	1	10	2	0	0	8	164	PPE	Yes
**90**	Positive	F	56	Nurse	Single	Never	No	21.88	56	1.6	No	No	No	No	10	0	1	10	2	0	0.4	8	0	PPE	No
**91**	Positive	F	36	Nurse	Widowed	Never	Yes	25.71	70	1.65	No	No	No	No	10	0	2	10	0	0	0	12	28	PPE	Yes
**92**	Positive	M	37	Nurse	Married	Never	Yes	36.33	105	1.7	Yes	No	No	No	10	0	0	10	2	0	0	12	30	PPE	Yes
**93**	Positive	F	41	Nurse	Single	Never	Yes	29.73	79	1.63	No	No	No	No	10	0	0	10	2	0	0	12	37	PPE	Yes
**94**	Positive	M	35	Nurse	Married	Previous	Yes	43.50	117	1.64	No	No	No	No	5	2	1	20	0	0	0	12	0	PPE	No
**95**	Positive	F	34	Nurse	Married	Never	No	35.36	101	1.69	No	No	No	No	10	0	1	10	0	0	0	8	0	PPE	No
**96**	Positive	F	27	Nurse	Single	Never	Yes	26.73	80	1.73	No	No	No	No	10	3	2	20	2	0	0.2	12	270	PPE	Yes
**97**	Positive	F	40	Nurse	Single	Never	Yes	23.44	60	1.6	No	No	No	No	10	1	1	50	0	0	0	8	7	PPE	Yes
**98**	Positive	F	31	Nurse	Married	Never	Yes	41.55	96	1.52	No	No	No	No	3	2	0	3	2	0	0	7.5	220	PPE	Yes
**99**	Positive	F	30	Nurse	-	Never	Yes	-	-	-	No	No	No	No	-	0	1	-	0	0	0.2	8	0	PPE	No
**100**	Positive	F	40	Nurse	Married	Never	Yes	23.34	59	1.59	No	No	No	No	3	0	1	10	0	0	0	8	0	PPE	No
**101**	Positive	F	49	Administrative	Married	Never	Yes	29.07	85	1.71	No	No	No	No	20	0	0	20	0	0	0	7	0	PPE	Yes
**102**	Positive	F	38	Nurse	Married	Never	Yes	27.94	68	1.56	No	No	No	No	10	0	0	24	0	0	0.2	8	24	PPE	Yes
**103**	Positive	M	39	Nurse	Married	Never	Yes	29.06	83	1.69	No	No	No	No	4	0	0	6	2	0	0	8	51	PPE	Yes
**104**	Positive	M	25	Nurse	Married	Never	Yes	24.91	72	1.7	No	No	No	No	3	1	1	3	0	0	0	8	7	PPE	Yes
**105**	Positive	M	41	Doctor	Married	Never	Yes	40.75	115	1.68	No	Yes	No	No	3	0	0	10	1	0	0	8	240	PPE	Yes
**106**	Positive	F	30	Nurse	Married	Never	Yes	36.06	60	1.29	No	No	No	ypothyroidis	10	0	0	8	2	0	0	12	66	PPE	Yes
**107**	Positive	M	29	Nurse	Single	Current	Yes	29.32	95	1.8	No	No	No	No	0	1	2	1	0	0	0.4	12	65	PPE	Yes
**108**	Positive	M	30	Nurse	Single	Never	No	31.25	82	1.62	No	No	No	No	10	0	0	10	0	0	0	12	60	PPE	Yes
**109**	Positive	F	30	Nurse	Common Union	Previous	Yes	25.86	73	1.68	No	No	No	No	0	0	3	0	0	0	0	12	36	PPE	Yes
**110**	Positive	F	35	Nurse	Married	Never	Yes	23.01	56	1.56	No	No	No	No	3	0	0	2	0	0	0	12	28	PPE	Yes
**111**	Positive	M	21	Nurse	Single	Never	Yes	23.31	65	1.67	No	No	No	No	10	0	3	30	0	0	0.2	8	252	PPE	Yes
**112**	Positive	F	27	Nurse	Single	Never	Yes	26.99	64	1.54	No	No	No	No	10	2	1	15	2	0	0	8	70	PPE	Yes
**113**	Positive	F	29	Nurse	Married	Never	No	25.10	76	1.74	No	No	No	No	10	0	0	20	0	0	0.4	12	0	PPE	Yes
**114**	Positive	F	38	Nurse	Common Union	Previous	Yes	32.88	79	1.55	No	No	No	No	10	0	1	2	0	0	0.4	12	30	PPE	Yes
**115**	Positive	F	30	Nurse	Married	Never	Yes	24.03	60	1.58	No	No	No	No	10	0	2	20	0	0	0	12	20	PPE	Yes
**116**	Positive	F	32	Nurse	Single	Never	No	22.66	58	1.6	No	No	No	No	6	1	2	30	0	0	0	12	22	PPE	Yes
**117**	Positive	M	24	Doctor	Single	Previous	Yes	26.56	68	1.6	No	No	No	No	6	0	1	30	2	0	0.2	12	64	PPE	Yes
